# FADS2 Function Loss at the Cancer Hotspot 11q13 Locus Diverts Lipid Signaling Precursor Synthesis to Unusual Eicosanoid Fatty Acids

**DOI:** 10.1371/journal.pone.0028186

**Published:** 2011-11-30

**Authors:** Woo Jung Park, Kumar S. D. Kothapalli, Peter Lawrence, J. Thomas Brenna

**Affiliations:** Division of Nutritional Sciences, Cornell University, Ithaca, New York, United States of America; Paris Institute of Technology for Life, Food and Environmental Sciences, France

## Abstract

**Background:**

Genes coding for the fatty acid desaturases (FADS1, 2, 3) localized at the cancer genomic hotspot 11q13 locus are required for the biosynthesis of 20 carbon polyunsaturated fatty acids (PUFA) that are direct eicosanoid precursors. In several cancer cell lines, FADS2 encoded Δ6 and Δ8 desaturation is not functional.

**Methodology/Principal Findings:**

Analyzing MCF7 cell fatty acids with detailed structural mass spectrometry, we show that in the absence of FADS2 activity, the FADS1 product Δ5-desaturase operates to produce 5,11,14–20∶3 and 5,11,14,17–20∶4. These PUFA are missing the 8–9 double bond of the eicosanoid signaling precursors arachidonic acid (5,8,11,14–20∶4) and eicosapentaenoic acid (5,8,11,14,17–20∶5). Heterologous expression of FADS2 restores Δ6 and Δ8-desaturase activity and normal eicosanoid precursor synthesis.

**Conclusions/Significance:**

The loss of FADS2-encoded activities in cancer cells shuts down normal PUFA biosynthesis, deleting the endogenous supply of eicosanoid and downstream docosanoid precursors, and replacing them with unusual butylene-interrupted fatty acids. If recapitulated *in vivo*, the normal eicosanoid and docosanoid cell signaling milieu would be depleted and altered due to reduction and substitution of normal substrates with unusual substrates, with unpredictable consequences for cellular communication.

## Introduction

Several human cytogenetic and fine mapping studies have pin-pointed HSA 11q13 locus as a major hotspot for a number of human cancers [1], [2], [3], [4], [5]. Numerous genetic mechanisms have been reported, including 11q13 deletions, loss of heterozygosity, translocations and allelic amplification [3], [5]. The fatty acid desaturase cluster (FADS), encoded by genes *FADS1*, *FADS2*, and *FADS3*, localize within a 100 kb region on human chromosome 11q12–13.1 [6], [7]. FADS2 and FADS1 encode for critical enzymes for long chain polyunsaturated fatty acid (LCPUFA) biosynthesis, introducing double bonds between specific carbon atoms. Omega-3 (ω3 or n−3) and omega-6 (ω6 or n−6) PUFA are key nutrients linked to most of the diseases of humans, specifically cardiovascular (CVD), cancer, diabetes and metabolic syndrome, and are key structural components of neural tissue [8], [9]. The LCPUFA DGLA (20∶3n−6), ARA (20∶4n−6), EPA (20∶5n−3) and DHA (22∶6n−3) are precursors for cell signaling eicosanoids and their modification by biosynthetic inhibition of downstream metabolism are very valuable drug targets, for instance, cyclooxygenase and lipoxygenase inhibitors, and more recently docosanoids that are candidate drugs [10].

The FADS2 encoded Δ6-desaturase catalyzes the first and rate-limiting step in the biosynthesis of LCPUFA. In several cancer cells desaturation does not occur, which may be due to inactivation of the desaturase or of upstream (e.g. CoA production) or downstream (e.g. acyl transferase) steps. The reason for this defect has been suggested to be due to extensive chromosomal deletions, but no molecular evidence is available [11], [12]. The Δ8-desaturation substrates 20∶2n−6 and 20∶3n−3 are often detected, thus suggesting that the elongation step is functional [12], [13]. Until recently, the Δ8-desaturation substrates 20∶2n−6 and 20∶3n−3 were widely considered dead-end products, even though they are found in human plasma and red cells as well as other tissues. We showed the first molecular evidence that FADS2 catalyzes Δ8-desaturation for both of these substrates, representing an alternative route to LCPUFA biosynthesis in mammals where they are converted to eicosanoid precursor LCPUFA [14]. This pathway may be available when Δ6-desaturase activity is compromised.

The unusual loss of PUFA desaturase activity in several cancer cells prompted us to hypothesize that the primary defect resides in the desaturase itself, and not elsewhere such as in the activation of PUFA by CoA synthesis or synthesis of phosphoglycerides as acceptors of nascent LCPUFA. We confirmed that human breast cancer MCF7 cells do not possess any capacity for biosynthesis of LCPUFA because of absence of Δ6-desaturase activity [12]. Here we show the restoration of this metabolic defect by heterologous expression of FADS2, evidence of novel substrate specificity for FADS1, and competition between FADS1 and FADS2 for the same substrates, leading to the production of unusual LCPUFA which are not substrates for eicosanoid production.

## Results and Discussion

**Figure 1:**
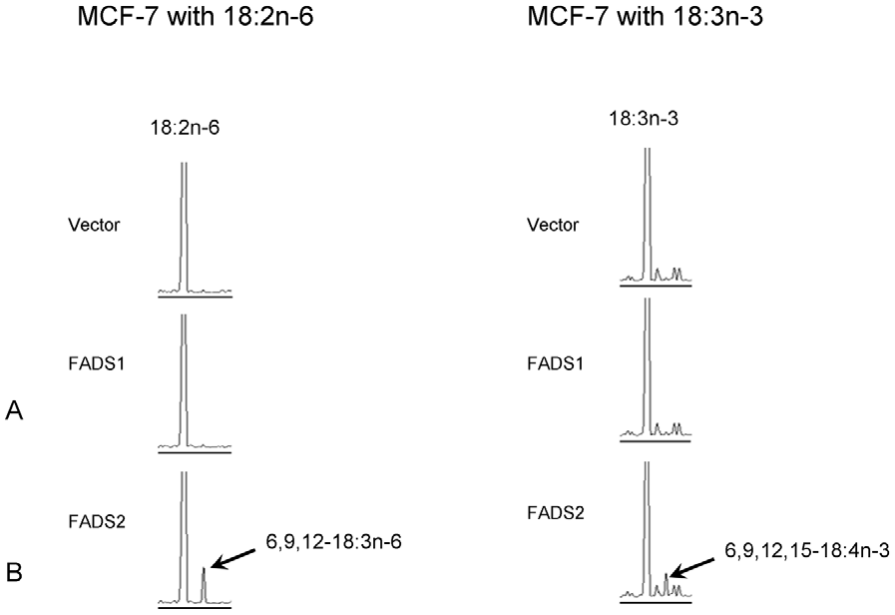
GC results of transfected MCF7 cells with18∶2n-6 and18∶3n-3 fatty acids. A: No product is seen in FADS1-transfected cells. B: FADS2 transfected cells Δ6-desaturate 18∶2n−6→18∶3n−6 (9,12–18∶2 → 6,9,12–18∶2) and 18∶3n–3→18∶4n−3 (9,12,15–18∶3 → 6,9,12,15–18∶4).

FADS2 and FADS1 transiently transfected MCF7 cells were probed for evidence of bioactivity toward 18∶2n−6 and 18∶3n−3. FADS2 transfected cells showed activity towards both the substrates; gas chromatography-covalent adduct chemical ionization tandem mass spectrometry (GC-CACI-MS/MS) confirmed the new peaks to be 18∶3n−6 and 18∶4n−3 respectively (Figure 1). As expected, no products were observed when either 18∶2n−6 or 18∶3n−3 were incubated with the FADS1 and the empty vector controls. These data provide the first unambiguous molecular evidence that the metabolic defect in these cells can be restored by replacing the rate limiting Δ6-desaturase enzyme encoded by FADS2.

**Figure 2:**
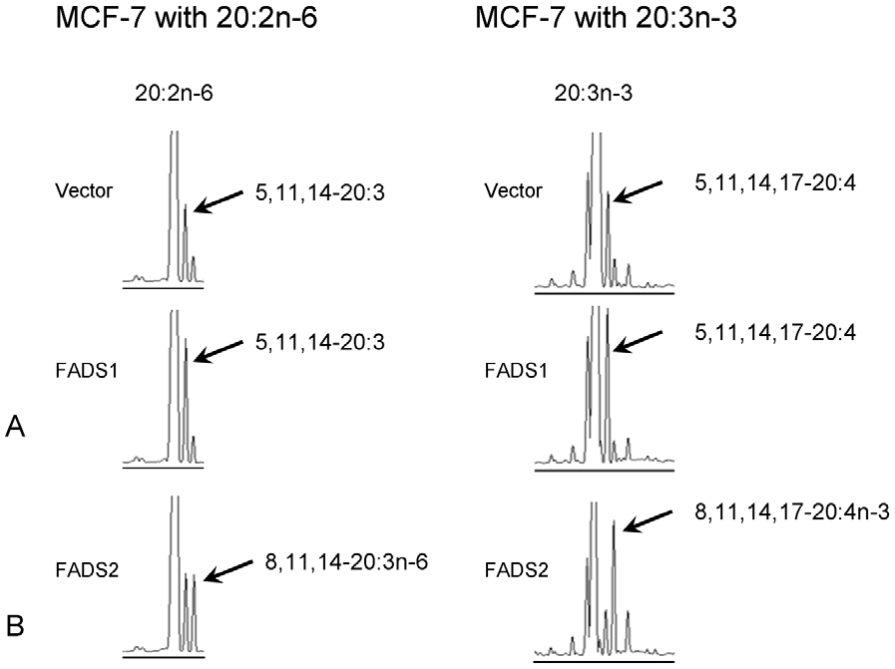
GC results of FADS2 transfected MCF7 cells with 20∶2n−6 (8,11–20∶2) and 20∶3n−3 (8,11,14–20∶3) fatty acids. A: FADS1-transfected cells Δ5-desaturate 20∶2n−6→5,11,14–20∶3 and 20∶3n−3→5,11,14,17–20∶4. B: FADS2 transfected cells Δ8-desaturate 20∶2n−6→20∶3n−6 and 20∶3n−3→20∶4n−3.

When FADS1-transfected cells were incubated with 20∶2n−6 and 20∶3n−3, CACI-MS/MS shows gain of synthetic function to generate two new butylene-interrupted PUFA peaks: 5,11,14–20∶3 and 5,11,14,17–20∶4, respectively (Figure 2). These two LCPUFA are analogues to arachidonic acid (5,8,11,14–20;4) and eicosapentaenoic acid (5,8,11,14,17–20∶5), respectively. Our data demonstrate that in the substantial absence of Δ8-desaturase activity (FADS2), the Δ5-desaturase (FADS1) operates on 20∶2n−6 and 20∶3n−3. Δ6-desaturation (FADS2) is normally about 10-fold more active than Δ5-desaturation (FADS1), thus it is plausible that during cell transformation FADS1 activity would persist when FADS2 is inactive. However, the preferred substrates for FADS1 are not essential fatty acids (18∶2n−6 and 18∶3n−3), but their elongation products 20∶2n−6 and 20∶3n−3. FADS1 acting on these substrates generates unusual butylene-interrupted carbon products and our results show that these rare fatty acids may be produced in cancer cells. This is the first molecular evidence showing novel FADS1 substrate specificity for 20∶2n−6 and 20∶3n−3 fatty acids. Moreover, FADS2-transfected cells demonstrate conclusively that the *FADS2* gene product Δ8-desaturates 20∶2n−6 and 20∶3n−3 to 20∶3n−6 (DGLA) and 20∶4n−3 (ETA) respectively, equivalent to our results in heterologously transformed yeast [14].

Moreover, while it is well known that both n−3 and n−6 PUFA substrates compete for same enzymes, our current data show FADS1 and FADS2 competing for the same substrates (Figure 2). The net consequence is the substitution of the inactive 5,11,14–20∶3 and 5,11,14,17–20∶4 for ARA (5,8,11,14–20∶4) and EPA (5,8,11,14,17–20;5), respectively, with unpredictable consequences for eicosanoid-mediated cell-cell paracrine signaling. Dysregulation of eicosanoid signaling is linked to tumor development, angiogenesis and metastasis in animal models [15]. A double bond at position 8 is required for cyclooxygenase (COX), lipoxygenase (LOX) and thromboxane biosynthesis, and thus the absence of the double bond at position 8 renders 5,11,14–20∶3 and 5,11,14,17–20∶4 inactive as substrates for biosynthesis of most eicosanoids [16], [17], [18]. Moreover, the possible action of the butylene-interrupted PUFA as competitive inhibitors of eicosanoid biosynthetic enzymes is unknown, as is the activity of other key eicosanoid synthetic enzymes (e.g. cytochrome P450) whose actions on normal parts of the unusual fatty acids would result in products with unknown activit(ies).

**Figure 3:**
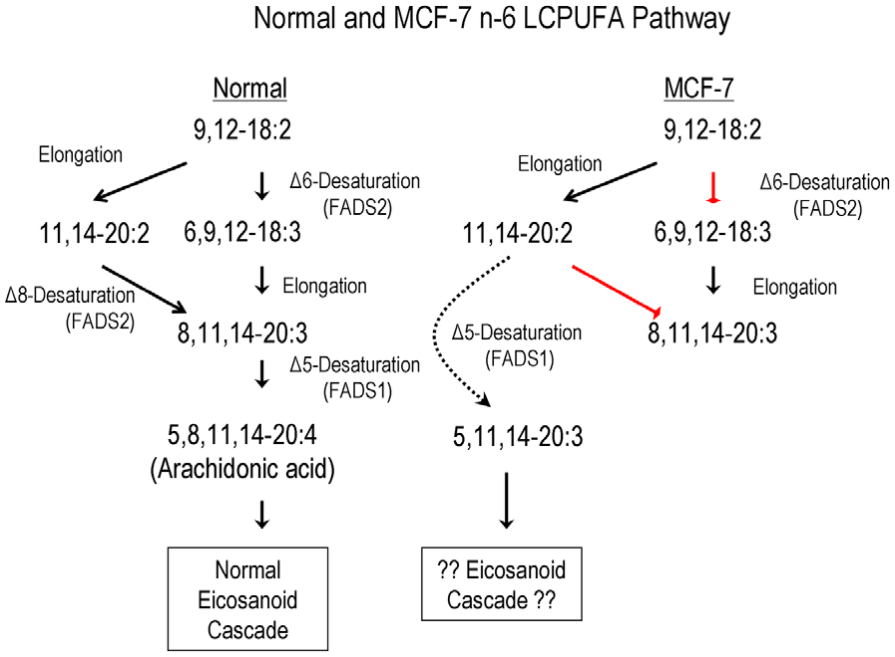
n−6 LCPUFA pathway in MCF7 cells. Δ6- and Δ8-desaturation steps are absent in MCF7, leading to Δ5-desaturation of 11,14–20∶2 to 5,11,14–20∶3 when FADS1 is functional. The analogous pathways for n–3 LCPUFA convert 11,14,17–20∶3→5,11,14,17–20∶4 (not shown). FADS2 is also believed to be involved in C22 LCPUFA biosynthesis (not shown).

FADS genes, encoding enzymes required for PUFA biosynthesis arose evolutionarily by gene duplication events. Despite key importance of FADS2 and the loss of its function in several cancer cells, the molecular details and consequences of FADS2 loss has not been described or investigated. FADS2 is known to have activity towards at least seven substrates (18∶2n−6, 18∶3n−3, 20∶2n−6, 20∶3n−3, 24∶4n−6, 24∶5n−3, 16∶0). The loss of FADS2-encoded activities in cancer cells completely shuts down the classical and alternative PUFA pathways, eliminating eicosanoid and docosanoid precursor biosynthesis from the plant-based PUFA linoleic and linolenic acids and thus limiting cell-cell signaling (Figure 3). Many studies have found strong associations between single nucleotide polymorphisms within the FADS gene cluster and complex phenotypes related to chronic disease, as well as to long chain PUFA levels [19], [20]. Further studies are needed to fully understand the consequences of FADS gene function loss and/or modulation based on SNPs in neoplasm. Early studies demonstrated that micro-cell mediated transfer of HSA11 into MCF7 cells reduced tumorigenicity, suggestive of potential tumor suppressor genes on this chromosome [21].

Our findings show that a primary molecular defect in MCF7 cells lies within the FADS2 gene causing loss of FADS2-encoded Δ6-desaturase activity. Compensation for FADS2 function by FADS1 leads to the production of 5,11,14–20∶3 and 5,11,14,17–20∶4, both primarily dead-end fatty acid products that cannot be precursors for most eicosanoids and are likely to be competitive inhibitors. The physiological significance of butylene interrupted PUFA in cancer cells calls for further detailed investigation. Our present results provide an impetus to better understanding the role of fatty acid desaturases, especially FADS2 as a tumor suppressor in neoplastic disorders.

## Materials and Methods

### Plasmid vector construction

The protein coding sequences of FADS1 (GenBank Accession# EF531577) and FADS2 (GenBank Accession# EU780003) were cloned into pcDNA3.1 expression vector using cDNA from neonate baboon liver tissue. The cDNA was obtained from banked samples drawn from a study approved by the Cornell University Institutional Animal Care and Use Committee (IACUC, protocol # 02–105). Analysis and comparison of amino acid sequence of baboon FADS1 showed 95% identities and 97% positives with human FADS1 (AF084558), whereas, baboon FADS2 showed 98% identities and 99% positives with human FADS2 (NM_004265). Earlier, we have shown novel Δ8-desaturation function using baboon FADS2 [14].

### MCF7 cell culture and fatty acid supplementation

For transfection experiments MCF7 cells were grown in MEM-α media with 10% Lipid-reduced FBS (media and serum obtained from HyClone) at 37°C in a humidified environment with 5% CO_2_. Human breast cancer MCF7 cells (American Type Culture Collection, ATCC, Rockville, MD) were the kind gift of Dr. Rui Hai Liu, Cornell University. The FADS1 and FADS2 constructs were transfected into MCF7 cells using Lipofectamine LTX (Invitrogen, USA) as per the manufacturer's recommendations. Twenty four hours after transfection, the MCF7 cells were supplemented with 100 µM of albumin bound 18∶2n−6, 18∶3n−3, 20∶2n−6 and 20∶3n−3 fatty acids and were incubated for additional 24 hours.

### Fatty acid analysis

After incubation with fatty acids, the cells were washed twice with 1XPBS and removed by trypsinization. The cells were harvested by centrifugation and the cell pellet was processed for lipid extraction. Fatty acid methyl ester preparation was carried out using a modified one-step method of Garces and Mancha [22]. Analysis was by gas chromatography-flame ionization detection (GC-FID) [8] and peak identification was confirmed by GC-covalent adduct chemical ionization tandem mass spectrometry (GC-CACI-MS/MS) [14], [23].
